# Use of a 6-miRNA panel to distinguish lymphoma from reactive lymphoid hyperplasia

**DOI:** 10.1038/s41392-019-0097-y

**Published:** 2020-01-03

**Authors:** Juanjuan Kang, Sisi Yu, Song Lu, Guohui Xu, Jiang Zhu, Na Yan, Delun Luo, Kai Xu, Zhihui Zhang, Jian Huang

**Affiliations:** 1 Center for Informational Biology, University of Electronic Science and Technology of China, 611731 Chengdu, China; 2 Sichuan Cancer Hospital & Institute, Sichuan Cancer Center, School of Medicine, University of Electronic Science and Technology of China, 610041 Chengdu, China; 3 Innovative Institute of Chinese Medicine and Pharmacy, Chengdu University of Traditional Chinese Medicine, 611137 Chengdu, China; 4 Research Center, Chengdu Nuoen Genomics, Ltd., 610041 Chengdu, China

**Keywords:** Tumour biomarkers, Predictive medicine

Lymphoma is a systemic malignancy originating from the lymphatic system, and it accounts for 3–4% of all tumors. In the United States, lymphoma is ranked 5th among the top causes of cancer deaths, and an estimated 80,500 new cases were diagnosed in 2017.^1^ Successful treatment relies largely on the correct diagnosis and subclassification of lymphoma in surgically excised biopsies based on cell morphology, immunophenotyping, flow cytometry, in situ fluorescent hybridization, and molecular diagnosis. However, the ability to distinguish between reactive lymphoid hyperplasia (RLH) and lymphoma is not an easy task. In routine pathology practice, lymph nodes in formalin-fixed paraffin-embedded (FFPE) sections show reactive changes more frequently than malignant features. Complicating the analysis, many reactive changes display atypical features and often mimic lymphoma, making these benign changes difficult to distinguish from malignant changes.^2^ New biomarkers that will address this challenge are urgently needed in clinical practice.

MicroRNAs (miRNAs) are endogenous small noncoding RNAs (22 nt) with regulatory functions. Increasing evidence has shown that miRNAs impact immune and inflammatory responses to bacterial infection.^3^ Meanwhile, many miRNAs play essential roles in lymphocyte differentiation and the malignant development of lymphoma.^4^ Furthermore, miRNAs are preserved well in many tissues, particularly in FFPE samples.^5^ Therefore, the discovery of miRNA biomarkers for the differential diagnosis of RLH and lymphoma using FFPE samples has become feasible.

To excavate candidate miRNA biomarkers, we obtained 31 relevant miR-seq datasets from the SRA database. This collection includes several major types of lymphomas, as well as B and T cells. We screened the most stably and highly expressed miRNAs by analyzing standard deviations and expression levels (Fig. 1b). Then, let-7f was selected as the reference gene as it exhibited the highest abundance and low variation, and the miRNA abundance in each SRA dataset was arbitrarily normalized to 100,000 copies of let-7f.

**Fig. 1:**
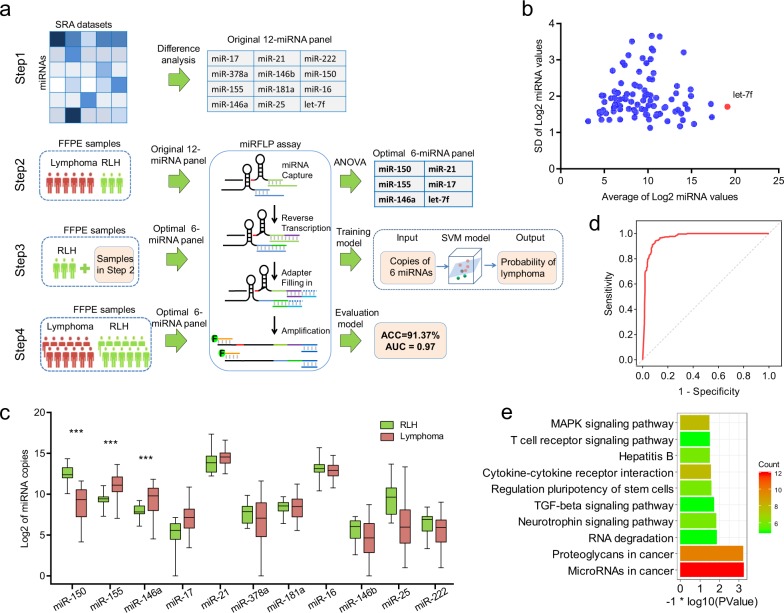
The construction of a 6 miRNA panel to distinguish lymphoma from RLH. **a** Flowchart of this study. **b** Selection of the stably expressed endogenous miRNAs as the reference. SD standard deviation. **c** Differential expression of 11 miRNAs normalized to let-7f levels in 144 FFPE samples. **d** ROC curve of the classifier using an independent sample set. AUC = 0.97. **e** The top 10 terms of the enriched KEGG pathway for target genes of miR-21.

Biomarker candidates were selected only from the top 50 miRNAs stratified by read depth to ensure assay sensitivity. According to the analysis of differentially expressed miRNAs, the upregulated miRNAs in lymphoma were miR-17 (95% CI: 882 ± 1164, *p* = 0.040) and miR-378a (95% CI: 6775 ± 7486, *p* = 0.095), while the downregulated miRNAs were miR-222 (95% CI: 518 ± 221, *p* = 0.030). Previous studies have reported important functions for miR-150, miR-155, miR-16, miR-146a, miR-25, miR-181a, miR-21, and miR-146b in lymphoma; therefore, we included these miRNAs in the set of candidate biomarkers. Ultimately, an original 12-miRNA panel was constructed, which comprised the 11 candidate miRNA biomarkers and let-7f as the reference.

The levels of the original miRNA panel in 124 lymphoma samples that covered the major lymphoma types and 20 RLH FFPE samples were measured using a microRNA-derived fragment-length polymorphism (miRFLP) assay, which is an extract-free single-reaction assay.^6^ After normalization to let-7f levels, the differential expression of 11 miRNAs is shown in Fig. 1c (Table S1). The power of 11 individual miRNAs to distinguish lymphoma and RLH was analyzed based on the *F* score obtained from the ANOVA.^7^ Then, the top five miRNAs (miR-21, miR-146a, miR-155, miR-17, and miR-150) were integrated to form an optimal 6-miRNA panel to distinguish lymphoma and RLH, with let-7f as the reference. An additional 20 RLH FFPE samples were collected, and then, the levels of the optimal miRNA panel in all 164 samples were measured again using the miRFLP assay. The detection results of the 164 FFPE samples containing 124 lymphoma and 40 RLH samples were used to train an artificial intelligence classifier using a support vector machine.

An independent sample set containing 262 lymphoma and 375 RLH samples was used to evaluate the performance of the classifier using ROC curve and accuracy analyses. The 637 samples in the independent sample set were analyzed using the optimized SOP of the miRFLP assay. Since the independent sample set did not overlap with the training set, it can convincingly indicate the performance of this diagnostic method. The classification threshold was set at 0.5. If the score was larger than 0.5, the corresponding sample was classified as lymphoma. The classification results are shown in Table S2. The AUC of the artificial intelligence classifier based on the 6-miRNA panel in differentiating lymphoma from RLH was 0.97 (Fig. 1d), with an accuracy of 91.37%. In addition, the classifier displayed an unexpected performance in the identification of extranodal NK/T-cell lymphoma and in the potential to identify mantle cell lymphoma from other lymphoma types (Fig. S1). In all, the independent sample test illustrated the excellent performance of the classifier in distinguishing between RLH and lymphoma samples.

Functional enrichment analysis was applied to the target genes of the five miRNAs in the optimal panel. These target genes are mainly involved in pathways in various cancers, the MAPK signaling pathway, the TGF-beta signaling pathway, and hepatitis B (Fig. 1e, Fig. S2, Table S3). The MAPK and TGF-beta signaling pathways are both strongly associated with tumorigenesis. The former can interact with P53 and regulate proliferation and the cell cycle in tumors, including lymphoma. The latter plays important roles in hematopoiesis, and its deregulation may contribute to the pathogenesis of hematologic malignancies. Surprisingly, hepatitis B virus (HBV) infection can promote the progression of non-Hodgkin lymphoma, but the specific mechanism remains unknown.^8^ The result of pathway analysis provides a novel angle to uncover how HBV induces the occurrence and promotes the evolution of non-Hodgkin lymphoma.

In summary, our study describes a simple artificial intelligence classifier based on a six-miRNA panel that displays high accuracy in differentiating lymphomas from RLHs. With the features of error-free sample loading, carrier RNA protection, the ability to analyze various suitable sample types, and extraction-free and multiplexed miRNA determination, the use of this classifier combined with the detection method is well equipped for clinical applications. As many altered miRNAs have been developed as therapeutic targets,^9^ the miRNAs in this panel may also have great developmental prospects for further targeted therapy of lymphoma.

## Supplementary information

Supplementary Methods and Figure

Supplementary Tables
