# Supplementary material for: Use of a 6-miRNA panel to distinguish lymphoma from reactive lymphoid hyperplasia
Source: Signal Transduct Target Ther. 2020 Jan 3;5:2. doi: 10.1038/s41392-019-0097-y (PMC6946694; doi:10.1038/s41392-019-0097-y)
Supplement: Supplementary file 1 — Supplementary Methods and Figure [file 41392_2019_97_MOESM1_ESM.docx]

Supplementary Materials for

Use of a 6-miRNA panel to distinguish lymphoma from reactive lymphoid hyperplasia

Juanjuan Kang,^1^ Sisi Yu,^2^ Song Lu,^2^ Guohui Xu,^2^ Jiang Zhu,^3 4^ Na Yan,^4^ Delun Luo,^3 4^ Kai Xu,^3 4^ Zhihui Zhang,^2^ and Jian Huang^1^

^1^ Center for Informational Biology, University of Electronic Science and Technology of China, 611731, Chengdu, China

^2^ Sichuan Cancer Hospital & Institute, Sichuan Cancer Center, School of Medicine, University of Electronic Science and Technology of China, 610041, Chengdu, China

^3^ Innovative Institute of Chinese Medicine and Pharmacy, Chengdu University of Traditional Chinese Medicine, 611137, Chengdu, China

^4^ Research Center, Chengdu Nuoen Genomics, Ltd., 610041, Chengdu, China.

Correspondence: Jian Huang (hj@uestc.edu.cn) or Zhihui Zhang (13881889739@139.com)

**This PDF file includes:**

Materials and Methods

Fig. S1 to S3

Materials and Methods

## Study design

This retrospective study was conducted to develop a miRNA panel and an artificial classifier to distinguish lymphoma from RLH with satisfactory diagnostic specificity and sensitivity. The study was performed in three phases: discovery, development and validation.

In the discovery phase performed in July 2015, we searched the NCBI Sequence Read Archive (SRA) database using the terms “Lymphoma”, “B-cell”, “T-cell”, “Hodgkin” and “DLBCL”. Then, the potential miRNA biomarkers were screened to construct the original miRNA panel for further analysis.

In the development phase, levels of the original miRNA panel in 124 lymphoma samples which covered the major lymphoma types and 20 RLH FFPE samples were measured using an miRFLP assay (Table S4). The results were analyzed using ANOVA and the optimization of the original miRNA panel was completed to produce the optimal miRNA panel. An additional 20 RLH FFPE samples were collected, and then the levels of the optimal miRNA panel in all 164 samples were measured again using the miRFLP assay(Table S4). The detection results of the 164 FFPE samples containing 124 lymphoma and 40 RLH samples were used to train an artificial intelligence classifier using a support vector machine (SVM).

In the validation phase, we generated an independent FFPE sample set, which consisted of 262 lymphoma and 375 RLH samples(Table S4). The lymphoma samples were consisted of eight lymphoma types. All cases used in this phase were new and had not been examined in the development phase. The performance of the optimal miRNA panel and the classifier was evaluated using the metrics of accuracy and AUC.

## Mining the potential miRNA biomarkers based on SRA dataset

We downloaded all relevant miR-seq data from the NCBI SRA database. Human mature miRNA sequences were retrieved from miRBase v21. Using the miRBase miRNA sequences as templates, the read depths for each miRNA were generated using an in-house software called miRay. The read depths can represent the relative expression levels of designated miRNAs in a sample. The differences in miRNA expression between lymphoma samples and normal B cells were compared. Additionally, miRNAs related to lymphoma in previous studies were also considered to identify potential miRNA biomarkers.

## Collection of FFPE samples

The retrospective study was approved by the Ethical Committee of School of Medicine, University of Electronic Science and Technology of China. The FFPE tissue samples used for the miRNA expression analysis were retrieved from the tissue bank of the Sichuan Cancer Hospital & Institute, Sichuan Cancer Center, School of Medicine, University of Electronic Science and Technology of China. Diagnosis and immunophenotyping were performed on FFPE tissues according to WHO classification system by a pathologist at our institute. In the development phase, FFPE samples included 124 lymphoma and 40 RLH cases. In the validation phase, 637 FFPE samples were collected, consisting of 375 RLH and 262 lymphoma samples. All FFPE samples were archived between 2008 and 2017 at the Sichuan Cancer Hospital & Institute. The detailed subclassification information is shown in Table S4.

## Extraction-free miRNA quantification using a multiplexed miRFLP assay

Briefly, the extraction-free miRFLP assay was performed using the following steps. Tissues were lysed in a buffer containing proteinase K and 0.5% SDS. RNA lysates were diluted with RNA Solution Buffer (RSB) containing 10 ng/µl bacterial RNA at a ratio of 1:50. Designated miRNAs and dynamic miRNA references were mixed and measured together using the miRFLP assay.

***RNA Lysate by SDS-PK method:*** FFPE tissues were scratched into a 1.5 ml tube and treated twice with 1 ml of xylene at 55℃ 5 min. Samples were washed twice in 1 ml of ethanol (100%) each. Frozen tissues, cultured cells or deparaffined tissues were mixed in adequate amount of 1x SDS-PK buffer containing 20 ng bacterial RNA, 0.5% SDS and 1 ug/µl proteinase K. Mixtures was incubated at 55℃ for 1 hour, and then denatured at 90℃ for 20 min. After cooled on ice, lysates were centrifuged at 15,000 g for 10 min at 4℃. The supernatants were used for miRNA detection directly or stored at -20℃for up to 2 months.

***RNA quantitation:*** Qubit RNA assay kit (Thermo Fisher, Q32855) and Qubit 2.0 Fluorometer (Life Technologies) were used for RNA quantification. For samples with RNA quantification, bacterial RNA carrier was omitted from 1xSDS-PK lysis buffer. The RNA lysate was diluted 200-fold in RNase-free ddH_2_O to counteract SDS interference of RNA quantification and then, 10 µl of diluent was added into 190 µl of Qubit working solution, incubating at 25℃for 2 min. RNA concentration was determined by Qubit RNA assay kit following vendor recommended protocol.

***RNA and oligonucleotide synthesis:*** Intrinsic RNA copy standard (iRCS), miRNAs, PCR primers, biotin-labelled omega primers and 3´ adapters in HPLC grade were purchased from GenScript, Nanjing, China. The sequence information of each oligonucleotide is listed in Table S5.

***miRFLP assay:*** We modified the original miRFLP assay to quantitate the absolute concentration of miRNAs from tissue lysates directly. First, 1 µl of tissue lysate was diluted in 49 µl of sterile H_2_O containing 10 ng/µl bacterial RNA. 1 µl of diluted tissue lysate was mixed with 7 µl of lysis buffer containing 2.5xMMLV RT buffer (Takara). The mixture was incubated at 75℃ for 3 minutes. After cooling to 4℃, 2 µl of various copies of iRCSs and 0.1 µl of RNase Inhibitor (RI, New England Biolabs) were added to the lysate. Next, 2 µl of probe mix containing 10 nM of omega probe for selected miRNAs and iRCSs was added to the mixture. Hybridization was performed on a PCR block with a 5-minute pretreatment at 55℃, which was then subjected to 10 cycles of 1 minute incubation at 55℃ and 5 minutes at 20℃. The reaction was held at 4℃ for 20 minutes. Then, 2 µl of enzyme mix containing 0.5 ml of MMLV reverse transcriptase (Takara) and 50 nM of dNTP (Sigma-Aldrich) was added to each hybridization reaction. Reverse transcription reaction was performed at 20℃ for 20 minutes and then at 37℃ for 5 minutes and 40 cycles of 15 second incubation at 55℃ and 1 minute incubation at 4℃, 20℃, 37℃, respectively. The reaction was stopped with 5 minutes of incubation at 85℃ and held at 4℃.

Subsequently, cDNA extension was completed with noise-reduction 3´ adapter as described in Table S5.

Next, cDNA fragments were purified with 1 µl of MyOne Streptavidin C1 (Life technologies) and washed once in 1x B&W Buffer before eluted in 10 µl of distilled water. Then, cDNA templates were amplified by competitive PCR as described in previous report. Final PCR products were diluted 1:100 in 1x TE and analyzed using an ABI 3730xl DNA analyzer at QingKe Biotechnologies, Chengdu.

The omega primers, iRCSs and 3´ adapters used for miRNA measurement were listed in Table S5. A string of unpaired nucleotides, in this case: aatttaa, was synthesized at the 3´ end to stop the priming feature of the 3´ adapters. Only the secondary probes derived from designated miRNAs or RNA fragments make perfect matches to the corresponding 3´ adapters to ensure further PCR amplification of desired fragments. Thus, mismatch signals were removed or identifiable by fragment lengths.

The final miRFLP PCR amplicons were labelled with fluorescent 5-Fam and the sequences and expected fragment lengths were shown in Table S6. The chromatogram plots of miRFLP fragment analyses were exemplified in Fig. S3.

***Optimized SOP of miRFLP assay:*** 1. Samples were treated with proteinase K in 0.5% SDS containing bacterial RNA. 2. Endogenous let-7f normalization was used instead of RNA quantification. 3. A mixture of synthetic RNA oligos was used as a quantification reference for miRNA copy calibration.

## Construction and validation of the SVM classifier

SVM was adopted to construct the classifier of lymphoma and RLH, based on miRFLP test results The LIBSVM2.84 package was downloaded from http://www.csie.ntu.edu.tw/~cjlin/libsvm. The radial basis kernel function (RBF) was selected to perform the prediction. In the SVM operation engine, two parameters (the error penalty factor C and kernel function parameter gamma) were optimized using the grid search approach. The accuracy and AUC metrics were applied to measure the performance. Five-fold cross-validation was used to assess the performance of the training model. The final classifier was validated with an independent sample set.

## Statistical analysis

The Wilcoxon test and ANOVA were performed using IBM SPSS (Statistical Package for the Social Sciences) version V19. The ROC curves and AUC were calculated using OriginPro 8 software.

## Pathway analysis

The targets of 5 miRNAs (miR-21, miR-146a, miR-155, miR-17 and miR-150) in optimal miRNA panel were recognized by the ENCORI, a new version of starBase. The mRNA targets should be predicted by at least 4 programs among PITA, RNA22, miRmap, DIANA-microT, miRanda, PicTar and TargetScan and supported by at least 1 Ago CLIP data. For target sets of each miRNA, we used DAVID to analyze the enriched KEGG pathway. The top 10 of enriched KEGG pathways were shown in Fig. S2 and Table S3.


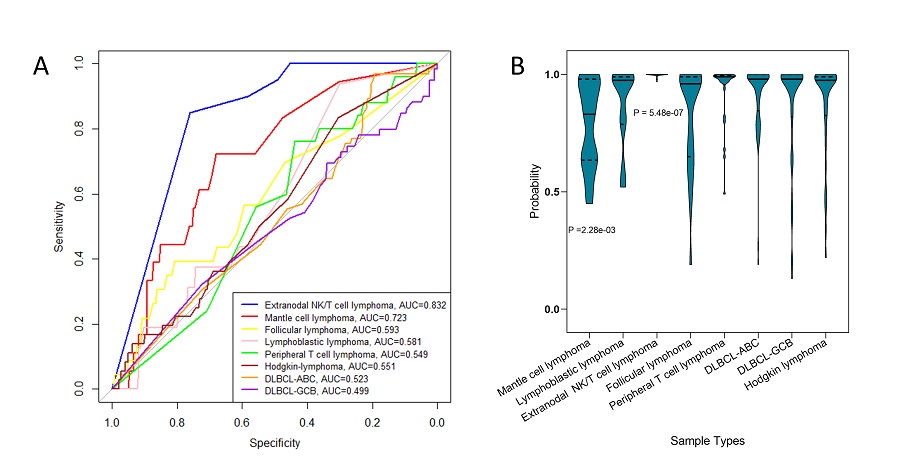


Fig. S1. The performance of classifier for identifying lymphoma types.

A ROC curves of classifier for identifying types of lymphoma. B The violin plots for predict scores in lymphoma types (Wilcoxon test).


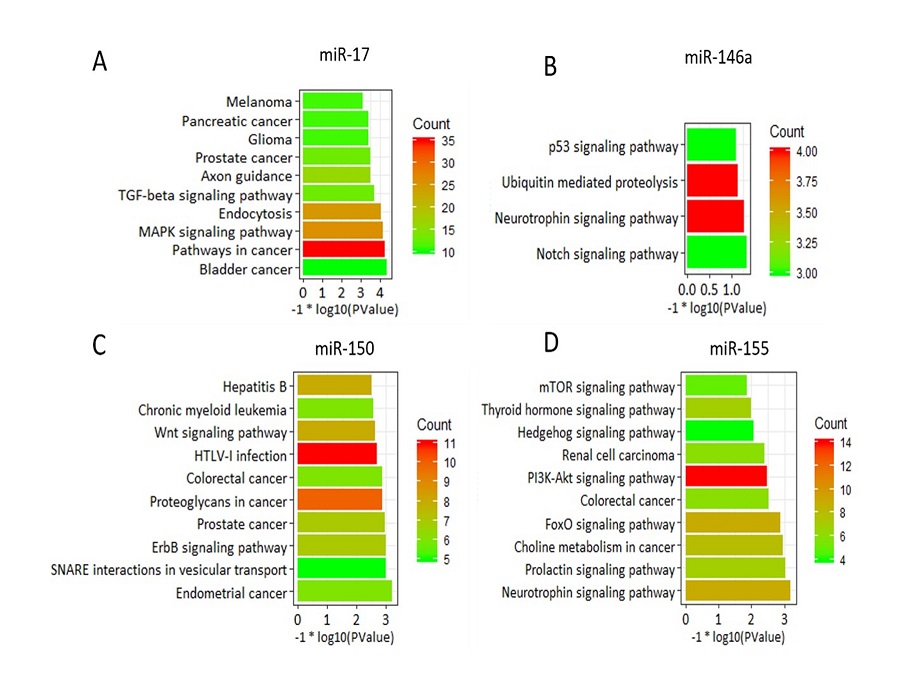


Fig. S2. The enriched KEGG pathways for target genes of miR-17, miR-146a, miR-150 and miR-155.

A The top 10 terms of enriched KEGG pathways for miR-17 target genes. B The enriched KEGG pathways for miR-146a target genes. C The top 10 terms of enriched KEGG pathways for miR-150 target genes. D The top 10 terms of enriched KEGG pathways for miR-155 target genes.


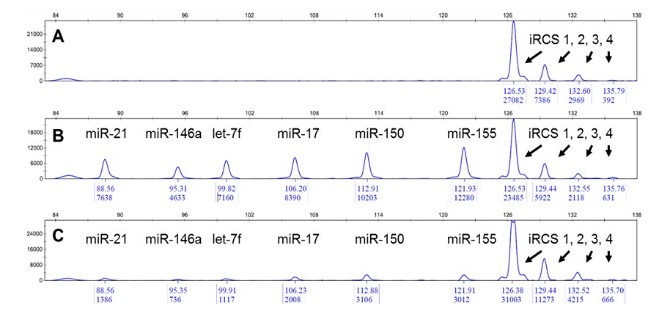


Figure. S3. Chromatogram views of 6-miRNA detection by miRFLP assay.

The graphic views of ABI 3730xl test results illustrated for various amounts of RNA samples. A 10 ng bacterial RNA tested as no template control. B 500,000 copies of miRNA reference mixture. C 18,519 copies of miRNA reference mixture. MiRNA reference mixture contained equal copies of miR-21, miR-146a, let-7f, miR-17, miR-150 and miR-155 respectively.
